# Parental Production of Child Sexual Abuse Material: A Critical Review

**DOI:** 10.1177/15248380231195891

**Published:** 2023-08-31

**Authors:** Michael Salter, Tim Wong

**Affiliations:** 1 University of New South Wales, Sydney, Australia

**Keywords:** sexual abuse, sexual exploitation, trafficking, child sexual abuse material, incest, cybercrime, internet

## Abstract

The aim of this review is to summarize the available empirical research on parental production and to explore the discursive positioning of parental perpetrators within scholarship on child sexual abuse material (CSAM). Academic databases were searched using a combination of relevant terms, and the review was expanded as new terms were identified. The review identified 66 scholarly articles, papers, or books that referred to parental production of CSAM published since 1970. To explore how parental offenders have been positioned within this literature over time, the review is presented according to a chronological summary, drawing out key themes and empirical insights. The review showed that parental CSAM production is common, more likely to involve pre-pubescent victims, more severe abuse, female as well as male perpetrators, and produces high-demand illegal content with serious long-term sequelae. However, the review found that the focus of child trafficking and sexual exploitation scholarship on “commercial” and profit-driven abuse has marginalized and obscured parental CSAM production as a serious policy challenge. These findings warrant a reorientation of research, policy, and practice approaches to technology-facilitated child sexual exploitation, as well as a reflection on the resistance of researchers and policymakers to acknowledging the problem of family-based sexual exploitation.

## Critical Findings Summary Table

- Parental figures are a significant group of producers of child sexual abuse material (CSAM).
- Parentally produced CSAM is more likely to involve the victimization of pre-pubescent children and more severe abuse.
- While parental CSAM production is typically initiated by a male offender, such as a biological father, stepfather, or mother’s partner, it often involves biological mothers who facilitate the exploitation of their children.
- Parental CSAM producers are often involved in networks of offenders who engage in organized, sadistic, and sometimes ritualistic abuse.
- Research into CSAM, exploitation, and trafficking often assumes that perpetrators are extrafamilial and their motives are commercial or profit driven. Acknowledgment of parental CSAM perpetration is inconsistent and fragmented.

## Implications for Research, Policy, and Practice

- Research studies into child maltreatment should ask specific questions about CSAM production to generate robust data on CSAM victimization and perpetration prevalence and scenarios.
- Research into CSAM, exploitation, and trafficking should acknowledge that parents are common perpetrators, particularly in cases involving prepubescent children.
- Research into parental CSAM production should address questions of diversity and intersectionality, such as similarities and differences in parental exploitation between low-income or marginalized families and higher-income and cultural majority families.
- Research, policy, and practice approaches to CSAM should avoid artificial “online” and “offline” distinctions, and instead examine and acknowledge how technology mediates sexual offending against children.
- There is a need for specialist and targeted policy and law enforcement responses to family-based sexual abuse and exploitation, online and offline.
- Men who seek to father children or partner with women with children to exploit those children are a serious but overlooked group of child sex offenders who require forensic and law enforcement attention.
- Victims and survivors of parental CSAM production are a profoundly traumatized and high-needs group who require a specialist and comprehensive response.

Since the popularization of the internet in the mid-to-late 1990s, the problem of child sexual abuse material (CSAM) has been growing exponentially. In this article, CSAM refers to images and videos in which children are sexually abused and exploited. For the last 20 years, reports of CSAM to US authorities have increased by an average of 50% per year (Bursztein et al., 2019) to the point where nearly 29 million reports of suspected abuse images and videos were made to American authorities in 2021 (NCMEC, 2022). Reports of CSAM jumped further during the COVID-19 pandemic, with strong evidence that child abuse offenders increased their CSAM consumption, distribution, and production during this period (Dąbrowska, 2021). CSAM production occurs in a variety of scenarios, including sexual exploitation within families, child-focused institutions, and through community-based networks or online grooming (Salter, 2013). CSAM is distinctive harm to child victims and adult survivors due to the ongoing trauma of image and video distribution, as well as the significant safety implications as CSAM offenders frequently stalk and harass their victims (Salter & Woodlock, 2023).

Parents have a prominent position in efforts to prevent online child sexual exploitation. They are a key audience for cybersafety resources and campaigns (Third et al., 2014). Sexual abuse prevention programs have been developed that aim to build parental capacity to protect children online as well as offline (Patterson et al., 2022). While these initiatives are important, an evident focus on protective parents in online safety frameworks overlooks the cohort of children who are sexually exploited by parental figures (e.g., mother, father, stepparent, parent’s partner, or foster parent). Research with adult survivors of CSAM finds that parents and familial figures are frequently identified as CSAM producers (C3P, 2017; Gewirtz-Meydan et al., 2018), and research with law enforcement and welfare professionals has drawn similar conclusions (Gallagher, 2007; Sprang & Cole, 2018). The predominance of parental perpetrators is not a new phenomenon. CSAM content analysis finds that family and home environments have been the most common setting for CSAM production for the last 50 years (Salter & Whitten, 2022). With the advent of the internet, parentally produced content constitutes the most highly traded and in-demand CSAM online, with a distinct trend toward the more severe abuse of younger children (Salter & Whitten, 2022; Seto et al., 2018). Despite the frequency, severity, and harms associated with parental CSAM production, it has been overlooked in policy and practice responses to online child sexual exploitation, which mirrors an overarching policy reluctance to specifically address family-based sexual abuse despite expanding attention to extrafamilial offenders (Salter, 2016).

In light of the invisibility of parental CSAM producers within online child protection discourse and frameworks, this literature review has two aims: first, to draw together the empirical evidence of parental CSAM perpetration, and, second, to explore the positioning of parental production within CSAM scholarship. The article presents a chronological literature review, tracing key lines of argument about the role of parents in CSAM production, reviewing the strength of the available evidence, and identifying underlying assumptions and biases. The paper then discusses the major findings of the review, including the current state of knowledge about parental CSAM production, and critically analyses the characterization of parental perpetration within scholarship on child sexual exploitation, trafficking, and online offenses. The paper also calls attention to the continuity of parental CSAM perpetration evident from the pre-internet to the post-internet period, including themes of premeditated and sadistic abuse, and points to the conceptual and epistemological as well as political and practical obstacles to acknowledging and responding to the sexual exploitation of children by parental figures.

## Methodology

The literature review was guided by the question “How does the scholarly literature discuss the parental production of CSAM over time”? The search strategy was broad and flexible to capture all relevant scholarship and commentary. The review began in 1970 since there are very few publications on CSAM prior to this period. Academic databases were searched using the combination of the following terms: child sexual abuse images, child exploitation material, child sexual exploitation material, child pornography, child sexual abuse material AND parent*, mother*, father*, familial. All identified abstracts and book blurbs were reviewed to identify whether the source discussed or made reference to parental production. If the source did not refer to parental production, even in passing, then it was not reviewed. All sources were reviewed, and notes were made about their discussion and characterization of parental CSAM perpetration. The bibliographies of sources were reviewed to identify other relevant sources that were not uncovered by the search strategy. In total, 66 papers, book chapters, and books were identified as in scope for this review.

A key focus of this paper is on the discursive positioning of parental producers of CSAM, recognizing that this form of offending is addressed across academic, clinical, and professional literature in a range of disciplines. To explore how parental CSAM production has been positioned within CSAM scholarship over time, the search results were grouped by decade: 1970 to 1979, 1980 to 1989, 1990 to 1999, 2000 to 2009, 2010 to 2019, and 2020 to current. The review concluded in January 2023. The authors then wrote up a decade-by-decade summary of the reviewed literature with a focus on the key themes and research findings. These summaries were reviewed, and the first author developed an overarching narrative across the six decades of the review, observing shifts in terminology, emphasis, and framing, and extracting empirical data on CSAM perpetration. The emergence of key themes or terms during the review process necessitated additional nested searches into references to parental CSAM production in the context of “sex rings” (a key term in the 1980s literature), “commercial” sexual exploitation (a common term from the 1990s), and “familial trafficking” (which became increasingly common from the 2000s). In light of expanding empirical research findings over the last two decades, scholarship since 2000 has been presented in three key streams: (1) online offenders and criminal justice data, (2) anti-trafficking research, and (3) interviews and surveys with CSAM survivors.

## Chronological Overview

### 1970s

CSAM emerged as an academic and policy concern in the 1970s in the United States, as police and media investigations into child abduction and CSAM production triggered congressional hearings into child sexual exploitation and prompted a campaign of child protection law reforms (Donnelly, 1979; Pope, 1978). The first modern “child pornography” investigation occurred in 1973 and found that 27 boys had been sexually exploited and murdered by a network of offenders as part of the production of CSAM (US Attorney General, 1986, p. 599). In CSAM scholarship during this decade, estimates of the number of victimized children and the extent of CSAM production and distribution in the United States varied widely. Descriptions of CSAM production were often anecdotal and linked to police and clinical encounters with cases, or were reliant on presentations of the problem in media outlets and public inquiries. The claim is that most victims of CSAM are teenaged “runaways” (and therefore that most CSAM depicts adolescent victims exploited outside the family), but “there is some incidence of parents introducing their own children into pornographic modeling” (Payton, 1978, p.509) is a consistent theme throughout this period. Commentators frequently drew parallels between the sexual exploitation of children and the sexual exploitation of adults, with a particular focus on the victimization of teenaged “runaways” by so-called “pimps.” For example, in the FBI Law Enforcement Bulletin, McKinnon writes:

children of all ages are being procured and pandered by what are called “chicken pimps” to take part in prostitution and/or the manufacture of pornographic films for a price. (McKinnon, 1979, p.18)

However, McKinnon goes on to recognize that perpetrators of “kiddie porn” may be a “relative, neighbor, or a child already involved in the business” (p.18). Likewise, in Donnelly’s (1979, p.298) summary of the available evidence of CSAM at the time, he emphasizes that “[i]n the most extreme cases, children are coerced into posing by their own parents,” referring to cases in which adult “prostitutes” were accused of exploiting their own children. In his assessment of the adequacy of existing law to prosecute CSAM cases, Pope (1978, p.716) recognized that “some parents sold their own children into sexual and pornographic prostitution,” which was “an activity not contemplated by ordinary sex offenses.”

A small number of sources argued that parents have a significant or major role in the production of CSAM. Baker (1978, p.834) observed that “parents who allow their children to participate in sexually explicit activities are central figures in the child pornography process.” He quoted from a police officer who stated that “a constant rule seems to be that children under the age of nine are usually introduced to it (child pornography or prostitution) by their parents,” who were often CSAM victims themselves or may be exploiting their children to support a drug or alcohol addiction (Baker, 1978, p.818). He noted media reports of sexually abusive parents who abuse their children and “swap pictures with other incestuous parents,” as well as graphic letters from sexually exploitative parents published in pornographic magazines (Baker, 1978, p.818). His paper emphasized that CSAM legislation should be drafted with parental perpetrators in mind to ensure they are held accountable for the exploitation of their children.

Densen-Gerber and Hutchinson’s (1979) paper on the commercial sexual exploitation of children framed the problem primarily through the lens of parental perpetration. The authors argued that the public recognition of the problem of parental CSAM perpetration has been complicated by “confused and sympathetic” views of incest (p.61); that is to say, a tendency to view family-based sexual abuse as less serious or less harmful than extrafamilial child sexual exploitation. They went on to argue that CSAM producers frequently victimize their own children, and cited the frequency with which investigations for incest uncover CSAM “preceding and accompanying the assaults on the children” (p.62) which they argued is fueling the larger black market in CSAM. Against the “commonly stated belief that nude posing is harmless to the children” (Densen-Gerber & Hutchinson, 1979, p.62), they were at pains to establish the severity of the abusive acts depicted in CSAM as well as the insufficiency of existing laws to adequately prosecute cases of CSAM production and distribution.

### 1980s

The major strain of CSAM-related research in the 1980s occurred under the rubric of “sex rings.” This work was inaugurated by Burgess et al.’s (1984) review of FBI child sexual abuse case files from the 1970s, in which she and her coauthors provided a fine-grained analysis of multi-perpetrator, organized child sexual abuse based on police investigations and prosecutions. This work was the first systematic inquiry into child sexual exploitation based on more than investigative journalism, police, or clinical anecdotes. The “sex ring” literature combined clinical assessments of child victims with the known details of investigated cases, typically noting that familial offending played a role in CSAM production but that it was a rarity or incidental to sexual exploitation (Wild, 1989). For instance, Lanning (1989) published a report based on his experience as an FBI agent investigating “sex rings,” noting that some abuse groups involve the parents of the victims but arguing that “parents are not usually the abusers in child sex ring cases” (p. 10). Books by Ennew (1986) and Campagna and Poffenberger (1988) argued that most child “prostitution” and “pornography” involved teenage runaways but observed that some CSAM (particularly of prepubescent children) was made by parental figures.

During this same period, the mental health literature began to describe a cohort of child and adult clientele reporting sexual exploitation by parents. The burgeoning literature on dissociative disorders during the 1980s noted the presentation of clients who recalled parental sexual abuse as well as the production of “child pornography” (Fine, 1989; Kluft, 1987). Social workers and therapists also documented the overlap between incest and the manufacture of CSAM (Dominelli, 1986; Herrmann, 1987). These reports began to be countered by literature that claimed that there was no widespread demand for CSAM and that fathers were at risk of false allegations. An exemplar of this counter-scholarship is Stanley’s (1988) article “The child porn myth,” which claimed that the production and distribution of CSAM in the United States were “virtually eliminated” by the late 1970s (p. 295) and that efforts to investigate and prosecute child sexual exploitation are proceeding on “little or no evidence” (p. 297). He claimed that fathers were being prosecuted for CSAM offenses for taking innocent photos of their children in the bath or for naked photos that children make of themselves. Stanley would later be charged and convicted of CSAM production on a commercial scale (Cheit, 2014, p. 166).

### 1990s

There is relatively little scholarship on parental CSAM production during the 1990s, despite expanding concern about CSAM linked to the commercialization of the internet during this period, as well as some scholarly attention to “organized abuse” (i.e., the sexual abuse of multiple children by multiple adults, see Bibby, 1996). In one of the final papers to use the term “sex ring,” Hunt and Baird (1990) presented case studies of 10 children aged between three and five who had been sexually abused by multiple perpetrators who produced CSAM. Fathers were the primary offender in two cases. Their paper described the ways in which the recording of abuse amplified the “shame, humiliation, and powerlessness” of child sexual abuse and significantly compounded the trauma of their victimization (Hunt & Baird, 1990, p. 202). There was passing reference to parental CSAM production in the literature on complex trauma and dissociation. For example, Ross et al.’s (1991) review of the abuse histories of 102 adult patients who had been diagnosed with multiple personality disorder (now dissociative identity disorder) identified that the majority disclosed sexual abuse by parental figures, and 20.6% had been subject to CSAM production. Pediatric psychiatrists Nurcombe and Unützer (1991) presented a case study of a 5-year-old child who was removed from her parents due to medically assessed child sexual abuse, as well as other evidence of neglect and abuse, who disclosed sadistic and ritual abuse by her parents and others that included the production of CSAM.

The mid-to-late 1990s included key feminist contributions to debates on CSAM, noting that familial offending had been overlooked or sidelined in policy discussions and psychological research on CSAM offending. In their research report on child sexual exploitation, Kelly et al. (1995) drew on a range of sources including media and government reports to emphasize the participation of parents in CSAM production. They argued that parental exploitation has unique dynamics that require a specific policy and practice response. Kelly (1996) went on to criticize the exclusion of evidence of parental perpetration from policy debates. Commenting on an international seminar on sexual exploitation in Europe, Kelly (1996) noted:

there was marked discomfort at attempts to broaden the definition of sexual exploitation through reference to familial contexts in which child pornography is produced and children may be prostituted. (p. 45)

She observed that the focus of policy discourse on the commercial sexual exploitation of children prioritized CSAM production for financial gain, and overlooked sexual exploitation driven by other motives, such as sexual pleasure and offender access to a wider pool of child victims. Kelly (1996, p.45) also commented on the ways in which forensic psychology typologies created false distinctions between incest offenders and CSAM offenders, such that “investigations of ‘familial sexual abuse’ seldom involve either searches or questions [about CSAM],” and emphasized the importance of listening to testimony from CSAM survivors, many of whom attest to the overlap between incest and CSAM production.

This point is expanded upon by Itzin (1996, 1997) in her interview-based research with adult survivors of CSAM. She argued that CSAM production is a “part of all forms of intra-familial and extra-familial abuse and is itself a form of organised abuse” (Itzin, 1996, p.167). Drawing on her interviews with adult survivors of family-based child sexual abuse, she commented that “the existing definitions of child sexual abuse which ignore pornography and isolate paedophilia from incest, incest from extra-familial abuse and all of these from something called ‘organised abuse’, are misleading” (p.188), and explicitly challenged forensic psychological typologies which drew strict distinctions between men who have sexual relations with adults and men who sexually abuse children, and between incest offenders and CSAM offenders.

### 2000s

Until the 2000s, the underground and secretive nature of CSAM made it difficult to draw firm conclusions about the nature of CSAM production. However, intersecting developments from the early 2000s created a more conducive environment for research and data collection on CSAM production, including parental perpetration. The commercialization of the internet made visible and undeniable the propensity of child sexual abusers to network with one another and produce and exchange CSAM, generating new data on the problem (Jenkins, 2001). There has been an expansion since the early 2000s in anti-trafficking efforts in the United States, which has come to encompass child sexual trafficking, including CSAM production. More generally, from the early 2000s, there have been successive revelations of clergy and institutional abuse as well as the sexual abuse of children by high-profile individuals, which has driven public awareness and academic inquiry into the problem of child sexual exploitation (Salter, 2017). Three areas of scholarship relevant to parental CSAM production have developed over the last two decades and are described in separate sections below. The first draws on criminal justice data about CSAM cases generated through investigations and prosecutions for online offending. The second is auspiced by anti-trafficking research, and the third area involves the direct engagement of CSAM survivors in survey and interview research.

#### Online offenders and criminal justice data

Since the early 2000s, CSAM research has been dominated by a focus on online offending, linked to the unprecedented increase in CSAM availability in the advent of the commercialization of the internet. CSAM research based on law enforcement data in the United States, United Kingdom, and Australia has consistently identified a significant cohort of parental producers. For example, in the United States, analysis of law enforcement data on “child pornography” cases found that 25%–56% of offenders were family members or parents of the victims (Finkelhor & Ormrod, 2004; Quayle et al., 2008, p.40; Shelton et al., 2016). A study by Wolak (2015) examined US law enforcement data on technology-facilitated organized child sexual abuse and identified that over one-third of cases involved familial offenders. In comparison with extrafamilial cases, familial cases were more likely to involve CSAM production. A multi-method study of online child sexual abuse cases known to British law enforcement concluded that the majority of offenders were the fathers of victims, who used the internet to distribute CSAM and/or traffic their child to other men (Gallagher, 2007; Gallagher et al., 2006). An analysis of 82 prosecuted cases of parental CSAM production in Australia identified particular features of parental production: the majority involved paternal perpetration, 18% involved both the mother and father, and 10% involved a single maternal perpetrator (Salter et al., 2021).

These studies raise the prospect that “some biological or de facto fathers and stepfathers formed adult romantic relationships with the intention of producing or procuring children for exploitation” (Salter et al., 2021, p.14). While some parents may abuse biological children, single mothers may be targeted by male perpetrators who sought to use “her control over the lives of her children for his own sexual gain” (Elliott & Ashfield, 2011, p. 96), including coercing her into the co-production of CSAM of her children (Prat et al., 2014; Salter et al. 2021). One study of 98 women convicted of online sexual offenses in the United States found that of the 72% of women convicted of CSAM production offenses, 71% abused their own child and 77% had a male co-offender (Bickart et al., 2019, p.12). In these scenarios, women were likely to appear in the image committing a contact offense against their own child and were thus “herself a subject of the pornographic images, as well as a partner in their creation” and a co-abuser of the child (p.12).

An emerging parental production scenario involves women who produce CSAM of their children at the behest of online “boyfriends” or men who approach them on dating apps with the promise of a relationship (Salter et al., 2021). A large survey of almost 10,000 Australians who had used an online dating app or website in the past 5 years found that 9.4% had received a request to provide a photo of their own children or children they have access to (Teunissen et al., 2022). Almost half of that group (4.6% of the total sample) had been pressured to provide sexual images of their children. Importantly, research on prosecuted or known CSAM cases has found that parentally produced CSAM is highly valued and traded among online offenders (Quayle et al., 2018; Salter and Whitten 2022; Seto et al. 2018).

#### Anti-trafficking research

Significant law reforms in the United States from the early 2000s spurred expanded anti-trafficking and anti-exploitation activities that began to identify domestic victims of parental CSAM production. Scholarship on trafficking and child sexual exploitation has focused on abuse for financial profit or some other economic gain which, as Kelly (1996) noted, has prioritized extrafamilial perpetration and can inhibit recognition of parental offenders. Nonetheless, anti-trafficking and anti-exploitation scholarship since the 2000s highlighted the frequency of parental CSAM perpetration. For instance, in a National Institute of Justice report on commercial child sexual exploitation, Albanese (2007) focused on extrafamilial “traffickers” but note that “[c]hildren may be photographed as part of intrafamilial child sexual abuse” (p.7). A study of 10 government-funded anti-trafficking task forces in the United States found that all task forces had encountered parents who sexually exploited their children, including through CSAM production, but “there was a stated reluctance and/or lack of awareness to view such exploitation as sex trafficking” particularly since parental exploitation often lacked a clear financial motive (Smith et al., 2009, p.7).

In the anti-trafficking literature, studies with a strict focus on “commercial” exploitation (i.e., for financial gain) have found a lower prevalence of parental perpetration compared to those with a broader definition of trafficking. For example, in their analysis of US law enforcement data on “prostitution,” Wells et al. (2012) specifically reported cases involving the abuse of children by third parties for financial compensation. They identified that 26% of offenders were “family” or acquaintances of the child victim in cases where the internet was involved in the exploitation of the child, including the sale of CSAM. Research studies that treated CSAM production as synonymous with trafficking, regardless of whether the financial payment was present, identified a higher preponderance of parental offenders. Research with American child welfare professionals found that CSAM production was a regularly reported feature of child sex trafficking cases and that parents and family members were the most commonly identified perpetrators (Cole, 2018; Cole & Sprang, 2015). In their case review of 31 children identified as having been trafficked (i.e., victimized through “involvement in prostitution, pornography, strip dancing”), all but one had been trafficked by a parent, and 16 had been subject to CSAM production (Sprang & Cole, 2018, p.187). Reid et al.’s (2015) comparison of girls trafficked by family members and those trafficked by extrafamilial perpetrators found that those trafficked by relatives (the majority of whom were parental figures) were abused at a younger age, and subject to more severe forms of child maltreatment. An interview study with 260 survivors of “domestic minor sex trafficking” in the United States found that one in six reported being trafficked prior to the age of 12, and this group almost exclusively reported being trafficked by a parent (Thorn, 2018).

Literature on parental CSAM perpetration has continued to expand within trafficking scholarship. For instance, Raphael (2020) conducted four interviews with adults whom she describes as being “pimped” by their parents, identifying the overlaps between paternal sexual abuse, CSAM production, and organized and often sadistic abuse by networks of perpetrators. Pacheco et al. (2022) reported on 10 interviews with male victims of parental trafficking, noting that many of the survivors were reporting relatively well-organized and large-scale criminal organizations in which children could be “swapped” and purchased, and CSAM could be produced. Sadistic abuse was reported by all participants and one participant reported ritual abuse.

#### Research with CSAM victims and survivors

There is a rich vein of qualitative research with CSAM survivors beginning in the late 1990s with Itzin’s (1999, 2001) work in the United Kingdom, who drew on interviews with survivors to challenge the assumption that CSAM and child sexual exploitation were necessarily extrafamilial offenses. This argument was taken up by Scott (2001), who presented the first interview-based study with adults who describe ritual child sexual abuse (i.e., the misuse of rituals in the organized abuse of children, see Salter, 2012). Against the highly sensationalized accounts of ritual abuse that have dominated media coverage, Scott’s (2001) qualitative research highlighted that ritual abuse survivors are typically describing family-based sexual exploitation including parental CSAM production. This theme is further developed by Sarson and MacDonald (2008) in their work on child torture in Canada. They focused on the perpetration of sexual exploitation of girls by parental perpetrators and noted the ritualistic and sadistic abuse described by this victim cohort, and argued that “[b]eing pornographically photographed deepened the wounds” for victims of abuse (Sarson & MacDonald, 2008, p.430).

Over the last 15 years, qualitative, quantitative, and select clinical work that engages CSAM survivors in research have consistently identified the co-occurrence of parental CSAM production with organized child sexual abuse, including sadistic and ritual abuse. While these studies are methodologically diverse and engage research participants in different countries and via different means, they have shed light on consistent patterns of offender behavior that are less visible via law enforcement and agency data. Relevant studies include a survey of an online convenience sample of 150 CSAM survivors (C3P, 2017), interviews with 24 self-identified adult organized abuse survivors in Australia (Salter, 2013), a case review of 10 women in treatment for prolonged sexual abuse by their fathers in Australia (Middleton, 2013), and a survey of 165 self-identified ritual abuse survivors in Germany (Schröder et al., 2018). This literature describes the early initiation of severe abuse of long duration with significant mental and physical health impacts, in which parents facilitated CSAM production as well as other forms of sexual exploitation and torture. The ongoing sexual victimization of familial CSAM victims into adulthood is a repeated finding across these studies (e.g., C3P, 2017; Middleton, 2013; Salter, 2013).

In contrast to the focus on low-income families evident in trafficking research, interviews and surveys with CSAM survivors consistently identify exploitation occurring across the socioeconomic spectrum. One online survey of 150 adult survivors of CSAM found that, where only one offender was reported, fathers or stepfathers were the offenders in 42% of cases, but the proportion of parental perpetration increased to 82% for those survivors describing organized child sexual abuse (C3P, 2017). Participants in this survey came from a diverse range of backgrounds and described CSAM production in the context of lower-, middle-, and upper-class families. The comparatively wealthy status of some parental CSAM offenders was further evident in Middleton’s (2013) research with 10 Australian women presenting for mental health care and disclosing sexual abuse by their fathers in adulthood. Middleton (2013) found that, in all cases, their father and/or other family members had been involved in producing CSAM of the victim and facilitating their sexual abuse by other men outside the family. Middleton (2013) emphasized that all the abusive fathers were or had been gainfully employed, and the sample included the daughters of middle-class as well as “extremely wealthy” families (p.263). The socioeconomic diversity of parental CSAM production was emphasized in other qualitative studies that included participants from low-, middle-, and high-income families, identifying a propensity for parental producers to engage in organized, sadistic, and ritual abuse that transcends class boundaries (Itzin, 2000; Salter, 2013; Scott, 2001).

## Discussion

Empirical research over the last half-century finds that parental figures have an important role to play in CSAM production, particularly of young children. Warnings to this effect have been evident in scholarly publications since the late 1970s but were largely ignored for the following two decades due to a widespread and entrenched assumption that CSAM producers are largely extrafamilial. While the advent of the internet undoubtedly made CSAM more available, it brought new visibility and urgency to the problem and provided new sources of data. From the 2000s, analyses of CSAM investigations and reports, interviews with law enforcement and welfare professionals, and surveys and interviews with CSAM survivors all found that parental figures are a significant group of CSAM producers. Meanwhile, content analysis studies found that CSAM produced in home environments was common (Salter and Whitten, 2022) and material depicting the abuse of young children by their fathers is the most highly traded CSAM online (Seto et al., 2018). This work makes a number of important conclusions: parentally produced CSAM is more likely to involve more serious abuse, younger children, and the participation of female as well as male perpetrators, and this content is highly sought after by online offenders.

At the very dawn of research into online CSAM, Kelly and colleagues (Kelly, 1996; Kelly et al., 1995) were prescient in observing that an arbitrary focus on “commercial” and financially driven sexual exploitation was trivializing the problem of parental perpetrators. While research into parental CSAM production has expanded since the 2000s, a focus on extrafamilial perpetration remains evident in trafficking and child sexual exploitation scholarship, which are framed by an overarching concern with “commercial” or profit-driven abuse. Trafficking scholars repeatedly express surprise at the preponderance of parental perpetrators in the sexual exploitation of prepubescent children. For example, Cole (2018) reports a preponderance of familial perpetrators “somewhat unexpectedly” (p.430) while Thorn (2018) described CSAM production by parents as a “less familiar” form of trafficking. Trafficking literature has continued to refer to CSAM as “child pornography” and routinely refers to children as “prostitutes” and their parents as “pimps,” despite widespread recognition that such terms should not be applied to child victimization. One anti-trafficking report described children sexually exploited by their parents as born into “the life,” using archaic slang for prostitution (Thorn, 2018).

Such terminology suggests a lack of attentiveness to the unique characteristics and dynamics of child victimization in parental CSAM production. Furthermore, acknowledgment of parental CSAM production in scholarship on trafficking and sexual exploitation was often restricted to marginalized and low-income parents who were described as substance abusing and/or involved in prostitution (Reid et al., 2015). By contrast, research studies drawn from criminal justice data as well as qualitative and quantitative research with adult CSAM survivors have identified sexual exploitation within families whose sexual violence is camouflaged by what has been called a “pseudo-normal veneer” (Kluft et al., 1984) of apparent functionality. The evidence that children subject to parental CSAM production in these “pseudo-normal” families may continue to be sexually abused by parental and other figures into adulthood was an alarming finding that was noted by a range of studies in this review. This scholarship also foregrounded the physical, sexual, and emotional abuse and neglect experienced by this victim group and the psychological and psychosocial sequelae of parental CSAM perpetration, including severe and complex traumatization and dissociation.

Research with CSAM survivors has consistently documented more severe and intensive experiences of abuse than was evident in policing and criminal justice data, where specific details about the abuse experienced by children were scant. Qualitative and quantitative research with CSAM survivors point to a clustering of abuse experiences including parental perpetration, sexual exploitation/trafficking, and sadistic and ritual forms of abuse. It is important to recognize that such reports of parental sexual exploitation have been greeted with considerable academic and media skepticism, although this appears to be waning, in part due to the indelible evidence of offender deviancy provided by the internet (Salter, 2017). There are other obstacles to developing a coherent picture of parental CSAM production, including the proliferation of terminology, with parental CSAM production addressed under different labels and across parallel areas of scholarship that do not necessarily intersect or draw from one another. A consequence of the dispersed nature of discourse on parental CSAM production is that contemporary researchers into child trafficking and sexual exploitation are now rediscovering patterns of abuse (such as organized child sexual abuse, sadistic abuse, and ritual abuse) that were first identified and studied decades ago but have yet to be met with a specific policy and practice response.

A complicating factor in the development of more sophisticated responses to CSAM has been widespread but clumsy divisions of child sex offending and offenders into “online” and “offline” types. However, this review has emphasized the pervasive role that technology plays in child sexual exploitation, including the incorporation of technology into family-based sexual abuse, where images and videos of familial abuse are recorded and distributed online. Such common scenarios of abuse transcend simple “online” and “offline” dichotomies and instead call for conceptual frameworks that acknowledge the technological facilitation of child sexual abuse. Notions of “technology-facilitated” family, domestic and sexual violence are common in research on violence against women (Afrouz, 2023; Henry & Powell, 2018) and this review suggests that the field of child protection and the study of child sexual abuse should take a similar step.

There are practical challenges to naming and intervening in parental CSAM production. Efforts in the 1980s to develop specific policy and practice frameworks to detect and intervene in familial sexual abuse proved to be highly controversial, prompting child protection frameworks that have focused instead on the threat of extrafamilial sexual abuse perpetration, or on marginalized and low-income families where sexual abuse takes place alongside other forms of overt dysfunction (Salter, 2016). This asymmetrical focus on extrafamilial child sexual abuse has persisted into the internet era. As Lollar (2013) notes:

Instead of addressing the complexities of sexual abuse within families, the existing approaches to child pornography perpetuate an illusion of the typical child sex abuser, a “sexual predator” living completely outside of normal society. (p.376)

A picture emerges from this review of a sustained pattern of “motivated ignorance” (Williams, 2021) of parental CSAM production among scholars and policymakers, in which ample evidence of parental perpetration is available but marginalized and ignored within academic and policy discussions of the CSAM problem. Parental sexual exploitation is such a gross betrayal of the vulnerability of a child that, (Salter & Woodlock, 2023) posits, such victimization scenarios may induce defense mechanisms in bystanders such as denial, disbelief, and dissociation. From this perspective, the persistence of false assumptions that most CSAM producers are extrafamilial or that child sexual exploitation and trafficking is driven by commercial or financial motives can be understood as mechanisms through which knowledge of parental perpetration is kept at bay, on the margins of legitimated discourse and state action, despite parental figures constituting a major source of highly traded and in-demand CSAM. The findings of this review suggest that these defense mechanisms constitute an unresolved distortion in the field of knowledge about CSAM production and need to be acknowledged and dismantled to develop an evidence-based picture of the CSAM problem and necessary responses.

The review has some limitations due to available evidence. First, there is a lack of rigorous and reliable data on CSAM and online child sexual exploitation. Common child sexual abuse research methodologies such as retrospective victimization surveys have not asked questions about image production and CSAM victimization. The majority of children victimized in CSAM have not been identified by authorities (ECPAT & INTERPOL, 2018). Available evidence of parental CSAM production is drawn from retrospective self-report surveys and interviews with adult CSAM victims, criminal justice data, reviews of law enforcement and child protection cases, and interviews with relevant professionals. There is a clear need for more systematic research and data gathering in relation to CSAM and online child sexual exploitation.

Second, there are significant terminological ambiguities across the reviewed literature. CSAM is known by a range of different terms, and references to parental CSAM production were made in disparate literature, including on “sex rings,” “organized abuse,” “trafficking,” “juvenile prostitution,” and “commercial sexual exploitation.” These terms fell in and out of favor depending on the decade and the geographic location of the authors (e.g., “sex rings” and “organized abuse” literature is primarily British, while scholarship on the “trafficking” of children and “juvenile prostitution” is primarily North American), demonstrating a lack of consensus on the most appropriate terminology in the field. This myriad of terms reflected a range of underlying assumptions about the most common scenarios of CSAM production that were often contradicted by empirical research into parental CSAM production. The analytic strategy of the review has sought to incorporate these disparate terminologies, address background assumptions, and identify empirical insights and patterns.

Third, this review is limited to English language scholarship and hence it focuses on abuse taking place in English-speaking, high-income countries such as the United States, England, and Australia. It is well recognized that parents and family have a significant role to play in the sexual exploitation of children in middle- and low-income countries and burgeoning scholarship on the live streaming of child sexual abuse from South East Asia emphasizes the participation of parental figures in these offenses (Napier et al., 2021). Such CSAM perpetration scenarios were not captured by the search strategy of this review. Importantly, by focusing on parental perpetration in high-income countries, this review challenges racial and colonial assumptions that parental and familial child sexual exploitation is limited to the Global South (Scott, 2001). Instead, the review highlights that parental CSAM production remains an ongoing policy and practice challenge in comparatively wealthy jurisdictions.

## Conclusion

This review has found that parental figures have an important role to play in the production of CSAM but, despite the cumulative empirical evidence, this fact has proven to be elusive in CSAM, exploitation and trafficking research as well as policy and practice responses. While key areas of scholarship on trafficking and child sexual exploitation have been structured by assumptions that marginalize and overlook parental perpetration, they are increasingly recognizing the presence and unique dynamics of cases involving parental and familial offenders. However, trafficking and sexual exploitation literature retain a focus on perpetration on marginalized and low-income families, overlooking parental perpetration within the middle- and upper-class families who enjoy the “status shield” (Hochschild, 1983) of class and often racial and cultural majority respectability. Research findings show that parental CSAM perpetration occurs in diverse family backgrounds for a variety of reasons that can include commercial motives but also deviant sexual interests in the abuse and degradation of children. Scholarship on parental CSAM perpetration since the 1990s has identified recurrent themes of organized, sadistic, and ritualistic abuse, as well as long-term sexual servitude continuing into adulthood, which highlight the premeditated and dangerous characteristics of many parental perpetrators.

These research findings contradict the forensic characterization of incest offenders as situational, opportunistic, or less risky than extrafamilial offenders. Instead, these findings suggest that, in the same way that some offenders will seek a position within child-focused institutions to sexually abuse children, some offenders are creating or joining family structures with the same intention. From this point of view, the family can be understood as an institution that is vulnerable to co-option by child sex offenders, much like any other institution. This threat is particularly acute in relation to offenders seeking to sexually abuse and exploit pre-pubescent children. Online and offline child safeguarding and crime prevention frameworks highlight the importance of a “guardian” in protecting children from harm; however, the parental perpetrator seeks, occupies, and subverts the “guardian” position precisely because of the power that it grants him or her over children. Despite the significant risk posed to children by parental perpetrators, this review has surfaced apparent epistemic obstacles to the recognition of parental CSAM production and a distinct preference amongst academics and related professionals to locate CSAM production outside the family, often in scenarios already described in the literature on adult sexual exploitation (e.g., “trafficking” by a “pimp”).

Intervention in family-based sexual abuse and exploitation is fraught and complicated, particularly in cases where parental perpetrators have the social or economic capital to defend themselves against accusations of child maltreatment (Campbell, 2023). Generally, states have preferred to avoid proactive detection and intervention in family-based sexual abuse, since such actions have sparked significant controversy and backlash (Salter, 2016). However, as this review makes clear, the problem of CSAM production and distribution will not be resolved without acknowledging and addressing the problem of parental production. Indeed, the available evidence suggests that CSAM production is trending toward the more severe abuse of ever younger children, and the most likely producers of such content are parental figures (Salter & Whitten, 2022; Seto et al., 2018). It is therefore imperative that embedded assumptions within research and policy that marginalize or overlook parental perpetration are challenged, and that research is carried out into the dynamics and characteristics of parental CSAM production to inform the development of specific and effective prevention, early intervention, prosecution, and victim support responses.
